# Does ChatGPT enhance equity for global health publications? Copyediting by ChatGPT compared to Grammarly and a human editor

**DOI:** 10.1371/journal.pone.0342170

**Published:** 2026-02-05

**Authors:** Ella August, Rachel Gray, Zaria Griffin, Matilda Klein, Julie M. Buser, Kirby Morris, Tamrat Endale, Hana Teklu, Pebalo Francis Pebolo, Elizabeth Anderson, Frederique Laubepin, Yolanda R. Smith

**Affiliations:** 1 Department of Epidemiology, University of Michigan School of Public Health, Ann Arbor, Michigan, United States of America; 2 Center for International Reproductive Health Training (CIRHT), University of Michigan, Ann Arbor, Michigan, United States of America; 3 University of Michigan Summer Research Opportunity Program, Ann Arbor, Michigan, United States of America; 4 Greenhills School Advanced Research Program, University of Michigan, Ann Arbor, Michigan, United States of America; 5 Department of Obstetrics and Gynecology, St. Paul’s Hospital Millennium Medical College, Addis Ababa, Ethiopia; 6 Department of Obstetrics and Gynecology, Gulu University, Gulu, Uganda; 7 University of Michigan School of Public Health, Ann Arbor, Michigan, United States of America; 8 Department of Obstetrics and Gynecology, University of Michigan, Ann Arbor, Michigan, United States of America; National University of Malaysia Faculty of Education: Universiti Kebangsaan Malaysia Fakulti Pendidikan, MALAYSIA

## Abstract

English language copyediting poses significant barriers to global health authors in academic publishing. Editing is too expensive for most researchers in low-income countries, and large language models (LLMs) like ChatGPT may offer a cost-effective alternative. The technology, however, has been criticized for its biases and inaccuracies. In a preliminary, in-depth case comparison, we compared the number and quality of corrections made by U-M GPT, a secure, University of Michigan-hosted generative AI tool, to those from Grammarly and a human editor to text from two draft papers written by Ugandan sexual and reproductive health researchers. Overall, U-M GPT made about three times as many corrections compared to the human editor and about ten times more than Grammarly. U-M GPT was the least discriminating in terms of quality: only 61% (51/83) of its corrections were judged as improvements. Despite this, U-M GPT has advantages, such as a broad scope of correction types, fast turnaround, and no cost. Its disadvantages, which reflect shortcomings of LLMs more broadly, include the need for prompt engineering skill, careful review of corrections, and high environmental costs due to energy consumption. Additional concerns involve data privacy and content moderation policies that restrict discussions on topics deemed as sensitive; these included words related to sexual and reproductive health. Although LLMs could improve equity, efficiency, and productivity, several important issues should be considered when using the technology. Larger follow-up investigations are needed to confirm our findings. Authors using LLMs should consult journal guidelines and disclose their use.

## Introduction

Though English is the dominant language required for scientific discourse in academic journals, it is not the primary language of most researchers seeking to publish in these journals [1]. This mismatch creates a power imbalance that puts English as a second language (ESL) writers at a disadvantage in publishing their research. A recent study identified some of these disadvantages: ESL researchers spend 30–51% more time writing a paper in English compared to those for whom English is the first language [2]. Despite this additional labor, rejection due to grammatical errors is still a barrier to publication [3–8]. Financially, editing services are beyond reach for many such researchers. In a recent study, 59% of early-stage Columbian researchers reported paying for editing services, however premium editing cost nearly half of their average salary [4]. This disparity is significant because researchers from countries where English is not the primary language are underrepresented in the academic literature [9–12].

New technologies may help address these publication barriers and disparities. While embedded spell and grammar checkers within Microsoft Word or Google Documents and the free version of the Grammarly software (https://app.grammarly.com/) have been used for years, large language models (LLMs) present new opportunities that could potentially support greater equity in publishing. For example, while ChatGPT (along with other LLMs) has been critiqued for its inaccuracy, bias, and unethical orientation in scientific writing [13,14] its role in copyediting has been underexplored. Lechien et al. [15] compared corrections by ChatGPT with a human copyeditor on a small sample of papers written by ESL writers and reported that ChatGPT provided some helpful grammar-focused corrections. Another 2023 study [16] evaluated ChatGPT for academic writing editing but drew different conclusions. The investigators, who used writing samples from ESL researchers, summarized their results as disappointing: “ChatGPT was not designed as a language editing tool, and it does not excel in this capacity.”

Our study adds to this conversation by considering the copyediting potential of LLMs compared to Grammarly and a human copyeditor as potential supports to help authors get accepted for peer review at academic journals. We define copyediting as correcting errors in grammar, spelling, syntax, and punctuation, and ensuring that terminology and conventions are properly used. We also consider copyediting to include checks on the structure, organization, and the clarity of ideas presented. Finally, copyediting includes suggestions to improve readability, flow, and style (subsequently referred to as readability corrections) [17,18].

In a preliminary, in-depth case comparison, we classified the corrections made by U-M GPT (a secure, university-hosted generative AI [genAI] service) and compared them to a human copyeditor, as well as those produced by Grammarly. Additionally, we evaluated the process for generating corrections from each editor. As such, our study aimed to compare the quality of corrections, usability, accessibility, and affordability of all three editors; however, our assessments of ChatGPT were indirect because we used U-M GPT, which is not publicly available. Additionally, while the ideal study would investigate the impact of copyediting on acceptance of papers for peer review, this would be very difficult to do in practice. Our study is an initial step toward a greater understanding of the promise LLMs may offer to position researchers for publication.

## Materials and methods

In this observational study, we collected and analyzed text from draft manuscripts written by Ugandan sexual and reproductive health researchers. These investigators previously completed a group research and writing training program run by the Center for International Reproductive Health Training at the University of Michigan (CIRHT-UM) and Pre-Publication Support Service (PREPSS) [19–21]. We classified the corrections made by U-M GPT (see Table in S1 Table for categories), and compared them to a human copyeditor, as well as those made by Grammarly. Our U-M GPT and Grammarly pilot study results and lessons learned are presented in the S1 Text Supporting Information.

The free web-based version of Grammarly was used. This software does not sell user data, which is protected by security measures. (Grammarly’s privacy policy, as of September 22, 2023 and posted September 19, 2023, is here: https://www.grammarly.com/privacy-policy#sectionSingleColumn_4C6KOleBeBhbafKqrRoEsj.) They also have protections so only the users are able to read text they upload or write within Grammarly.

### Main study

To identify sample papers, on 25 July, 2024 we circulated an informational flier to researchers who had completed the CIRHT/PREPSS training program mentioned above [19–21]. Two Ugandan researchers gave their written approval (in emails, both dated 27 July, 2024) to use their draft manuscripts. One paper described a quantitative study and the other described a study that included both quantitative and qualitative data and analytic approaches. Both papers were shared as Microsoft Word documents. Revised versions of these papers were later published in peer-reviewed academic journals.

We evaluated one paragraph from each section (i.e., the introduction, methods, results, and discussion) of each paper including 4 paragraphs from each paper and 8 total paragraphs. We arbitrarily chose the second paragraph from each section of each paper to evaluate and also included the second table from each paper.

To maintain data privacy, we used a large language model (LLM) based on OpenAI’s GPT-4.o, deployed through a secure, university-hosted instance available exclusively to University of Michigan users (https://umgpt.umich.edu/), subsequently referred to as U-M GPT. At the time of analysis (October 2024), the specifications of GPT-4.o were as follows: a context window of 128,000 tokens and a knowledge cutoff of October 2023. The number of model parameters has not been publicly disclosed by OpenAI. These specifications reflect the capabilities of the underlying model and inform its performance limitations and potential in editing tasks. Further technical details can be found at: https://its.umich.edu/computing/ai/gpt-in-depth.

Our final U-M GPT prompt for the main study in October of 2024 is shown in the Box. Some genAI experts advise avoiding multifaceted prompts because models may struggle to address multiple requests simultaneously [22]. We included this single prompt to align with our goal of testing the feasibility of genAI for copyediting under real world conditions. We felt that negotiating multiple rounds of corrections would be unwieldy and judged this as an infeasible strategy.

**Box: Final ChatGPT prompt used for text analysis of two academic manuscript drafts written by Ugandan authors, 2024.**

“Copy edit this text from a scientific journal article so that it can be submitted to a peer-reviewed journal. Correct spelling errors, grammatical errors, capitalization, tenses, typos, punctuation, subject/verb agreement and other types of errors. Please also revise to improve clarity, readability, flow, and style, however, if the meaning of the text is unclear then do not attempt to revise the text, instead state that the text is unclear.”

However, to identify potential limitations in our single-prompt approach, we conducted a sensitivity analysis that is described below.

We fed the sample texts to U-M GPT one paragraph or table at a time because the paragraphs were from different parts of the manuscript and not intended to be sequenced together. After U-M GPT generated the corrected text, we created a track changes document in Word using the “compare” feature to display U-M GPT’s corrections. Grammarly corrections were generated in September of 2024 with the free version of the software and the parameters or “goals,” were set for an “expert” audience, “formal” formality, with “general” as the single “domain” (type of writing).

The human corrections were generated by a professional copyeditor who is part of the CIRHT/PREPSS training program [19–21] (all participants received copyediting). The copyeditor agreed to share her edits on two papers for the purpose of the study in May of 2024. We used two papers edited by her in September of 2024. She was not told in advance which papers would be used. Because the training program has a steady flow of manuscript submissions, it would have been very difficult for her to guess which papers were flagged for the study, particularly in light of the time gap between agreeing and the papers being assigned to her.

The goal of the copyediting in this program is to help authors get accepted for peer review at an academic journal. The copyediting guidelines were to correct spelling, grammar, punctuation and capitalization, to correct tense errors, subject/verb agreement, typos, and other types of errors. Readability was to be addressed by streamlining and shortening overly complex sentences and awkward usage. The copyeditor was directed to flag unclear text and request clarification as needed. She reported the length of time to edit each manuscript (not just the paragraphs evaluated in this investigation).

### Analysis

Author names were removed from the manuscripts by the lead author (EA) prior to group review and discussion. Due to the high volume of papers evaluated by program staff and the similarity in topic areas, the papers were not memorable (the lead author chose them but did not know the authors personally or remember training them).

We created categories (see Table in S1 Table) to classify the corrections from the three editors based on a modified version of Park et al.’s categories [23]. Compared to Park et al., who compared an AI grammar checker to a human rater [23], we used fewer categories describing “sentence-level” corrections such as different types of verb and noun errors. We classified each correction into its respective category. During analysis, we found that U-M GPT removed key information such as references and we added a category to describe this “correction” but did not include it in our total tally because it was added in an ad hoc manner. The lead author (EA) created preliminary classifications of each correction, and then a team of four authors (EA, RG, ZG, KM) reviewed and approved each preliminary classification; disagreements were resolved through group discussion.

### Sensitivity analyses

We conducted two sensitivity analyses. First, to identify potential limitations in our single-prompt approach, we conducted a sensitivity analysis that compared prompt chaining, wherein a bigger prompt is broken into smaller, focused sub-prompts [22] to our single prompt approach. Our methods and results are described in the S1 Text Supporting Information.

Second, to assess the similarity of edits from U-M GPT to the publicly available version, we fabricated a paragraph and requested copyedits from both versions of ChatGPT on the same day using the single prompt from our main analysis. To show the variability within version, we re-ran the request a second time in U-M GPT. Our methods and results are described in the S1 Text Supporting Information, Table in S3 Table, and Boxes in S2 File–S5 File.

### Ethical clearance

The parent study, assessing clinical research self-efficacy pre-post CIRHT intervention, was granted an exemption by the University of Michigan Institutional Review Board (#HUM00203642). Our current study received separate IRB approval (#HUM00253887) building off the exemption from the parent study. Written informed consent was obtained from all participants in the CIRHT parent study intervention (#HUM00203642), and explicit permission to use manuscripts for this study was provided by both participants in an email to the corresponding author.

## Results

It took the human copyeditor 3.75 and 4 hours to edit Papers 1 and 2, respectively, however we did not ask her to track the time it took to edit each paragraph and table because she was unaware of which papers would be included in the sample. U-M GPT generated corrections nearly instantaneously, however, creating a track changes document for each paper took roughly 30 minutes. The Grammarly corrections were also generated within seconds, and the user was prompted to accept or reject one change at a time. This part of the process took us approximately 5 minutes.

Whereas the human copyeditor provided corrections on the text and tables, neither U-M GPT nor Grammarly had the capacity to provide corrections on uploaded tables. U-M GPT conveyed this limitation as: “I’m sorry, but currently, I cannot directly receive file uploads. However, you can copy and paste the content of the table into the chat, and I can help you with the copyediting. Please provide the text, and I’ll assist you accordingly.” We did not paste each table cell as suggested because it was too unwieldy, and we felt it would limit the ability of U-M GPT to evaluate the table as a whole. The Grammarly software does not allow for table uploads.

**Fig 1:**
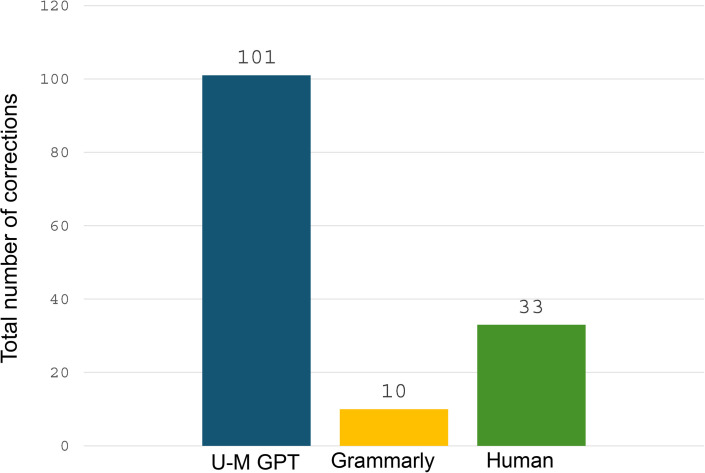
Total number of corrections by editor type, 2024.

In terms of overall suggestions, U-M GPT made about three times the number of corrections than the human editor and about ten times more compared to Grammarly (see Fig 1; a detailed breakdown of each correction type is shown in Table in S1 Table).

**Fig 2:**
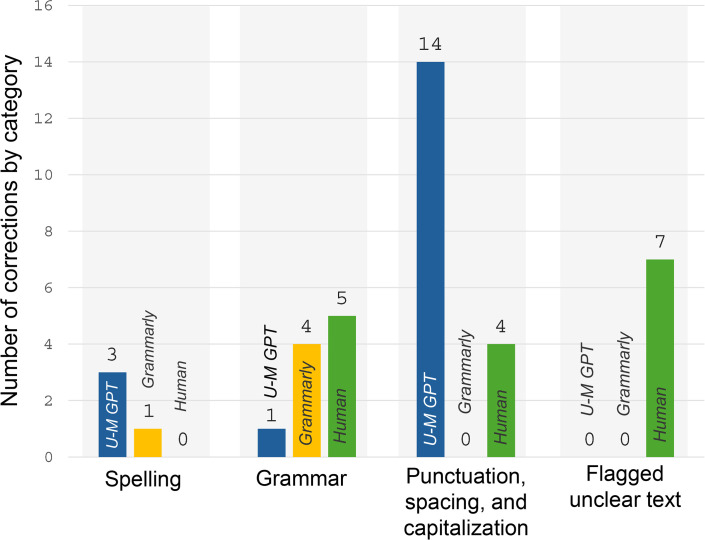
Spelling, grammar, punctuation, spacing, capitalization and flagged unclear text corrections by editor type, 2024.

The scope of the corrections varied by editor. While Grammarly made only two types of corrections (spelling and grammar), U-M GPT and the human editor each made three types of corrections. U-M GPT corrected spelling; grammar; and punctuation, spacing, and capitalization, while the human editor corrected grammar; punctuation, spacing, and capitalization; and also flagged unclear text (Fig 2). The editors made few spelling corrections; U-M GPT made three, Grammarly made just one, and the human editor did not make any. Each editor made grammar corrections, with the human editor making five, followed by Grammarly (n = 4), and U-M GPT (n = 1). Whereas U-M GPT made 14 punctuation, spacing, and capitalization corrections, the human made four and Grammarly did not provide any. Neither technology editor flagged unclear text, but the human editor flagged seven sections of unclear text.

**Fig 3:**
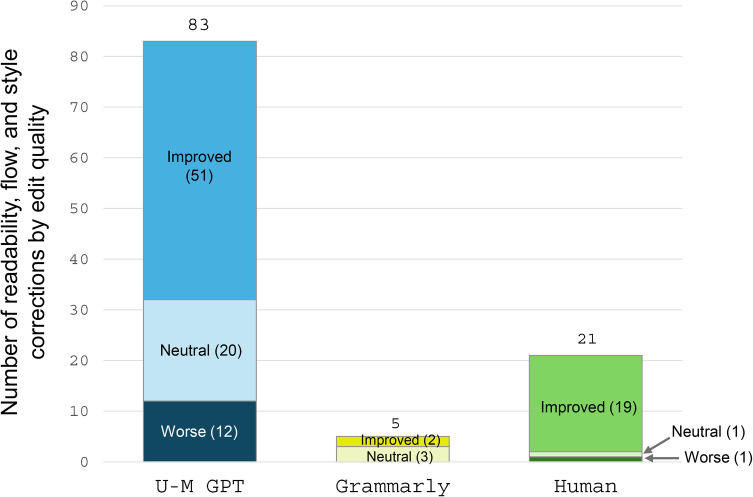
Readability and improvement of the text corrections by editor type, 2024.

In comparing readability corrections, U-M GPT was by far the most enthusiastic editor but the least discriminating in terms of quality (Fig 3). Whereas U-M GPT revised a total of 83 instances of text, less than two-thirds (61%; 51/83) of the corrections were judged as improvements. Fourteen percent (12/83) of U-M GPT’s readability corrections made the text less readable with poorer style or flow, and roughly a quarter (24%; 20/83) of the corrections were considered neutral. Grammarly only made five readability corrections; whereas two improved the text, three were judged as neutral changes that did not necessarily improve or worsen the text. The human editor made 21 corrections in this realm; most (90%; 19/21) improved the text, one was considered neutral, and one change was deemed worse than the original. Finally, U-M GPT removed 10 pieces of key information from one of the papers, including in-text citations and a reference to one of the tables (Table in S1 Table).

## Discussion

Our study addressed the important need for copyediting in helping authors get accepted for peer review at academic journals, with a particular emphasis on ESL researchers submitting to English language journals. Researchers with low English proficiency have reported greater challenges in preparing manuscripts compared to those with greater proficiency [24]. While there is no threshold for the “acceptable” number of language errors a journal will allow in an initial submission, reports identify that journal submissions are desk rejected based on language errors and “poor English.” [5–8] ChatGPT is being used in nearly every country across the world, including in low and middle-income settings [25] including for copyediting [13,15,16,26–28], representing a potential opportunity for accessible copyediting.

We investigated the feasibility of using U-M GPT in comparison to Grammarly and a human copyeditor. The subsequent discussion points are summarized in Table 1 to help readers appreciate the strengths and weaknesses of each editor type identified in this paper.

**Table 1:** Summary of strengths and weaknesses of each editor.

	U-M GPT	Grammarly	Human
Scope in types of corrections provided	Wide scope in types of corrections	Limited scope in types of corrections	Wide scope in types of corrections, but individualized, depending on knowledge and approach of editor
Ease of generating corrections	Requires some skill to create prompts	Easy	Easy
Accuracy of corrections provided	Moderate Accurate corrections mixed with neutral and poor corrections Revises unclear text based on its own interpretation rather than flagging it	High	High
Effort to view corrections	High Output does not show changes or compare to original document	Low Prompts user to accept or reject each change	Low Editor typically provides document showing changes
Effort to revise based on corrections	High Effort to view and review suggestions and decide whether to implement	Low Simple accept or reject actions	Moderate Effort to clarify text and respond to queries
Cost	Free version available Institutional cost for dedicated models such as U-M GPT^a^	Free version available Paid premium version available	Expensive or even unaffordable
Data privacy	Low: public version High: U-M GPT	High	High
Wait time for corrections	Negligible	Negligible	Variable
Accessibility based on topic	“Content moderation” filters prevent access in selected topical areas	High	High

^a^ A dedicated ChatGPT model refers to a version of ChatGPT offered exclusively to a particular institution or group and keeps data private.

The scope of correction types differed between editors, with the human editor offering the broadest and Grammarly providing the narrowest range of corrections. While all three editors provided corrections on spelling, grammar, punctuation, spacing, capitalization, as well as readability, only the human editor had the capacity to make suggestions on tables.

Additionally, only the human copyeditor flagged (rather than attempting to rewrite) passages in which the meaning was unclear for the author to evaluate and revise. Other types of corrections, such as checking the appropriateness of references are also currently either outside the scope of the technology-driven editors or would require an unwieldy workflow. While Grammarly cannot check a reference, ChatGPT could do this with guidance across separate steps. For example, ChatGPT could first be asked to “read” and summarize a paper, then identify whether a claim is supported by that paper. The output quality for such a request may be low [13,14], however, the technology editors may improve in this capacity soon.

In terms of the ease of generating corrections, requesting edits from a human copyeditor is quite simple, and we also found Grammarly to be very user friendly. However, an average researcher may not be highly proficient at prompt engineering, a necessary skill to produce high quality LLM outputs. As described in the S1 Text Supporting Information, we spent considerable time pilot testing different prompts, however we did not ask U-M GPT to focus on a single task at a time, for example, with a prompt chain approach advocated by some experts [22]. This would have required multiple rounds of requests and review, which seems infeasible for most researchers. We did test one paragraph with a prompt chain approach to get a sense of how U-M GPT’s corrections compared to a single prompt and found that the results did not substantially differ. The prompt chain approach was bulky and time-consuming, and given the time demands for a single-prompt approach are already substantial, the advantages of using the prompt chain approach are not obvious for copyediting based on our results.

There were similarities in the types of corrections that resulted from the three editing approaches. Simple corrections like spelling were not highly prevalent from any editor. This could be because Microsoft Word already provides spellcheck (both papers were provided to us in that software application; Google Documents has a similar feature). Thus, spelling corrections may not be an important need from AI or even human editors.

There were large differences in the number and type of corrections each editor made, particularly for the readability corrections. Though U-M GPT made numerous such corrections, about a quarter were judged as having a neutral impact on text quality, and 14% were judged to make readability worse. In a similar study that compared ChatGPT corrections to a professional editing service on four academic manuscripts, ChatGPT-4 corrected 86 of the 171 errors identified by a human editor. ChatGPT-4 proposed appropriate corrections on 84% of these 86 errors [15]. While ChatGPT provided a greater proportion of appropriate corrections in that study compared to ours, those authors focused more on subject/verb agreement, conjugation, spelling, and punctuation which are more clear-cut compared to readability corrections. Those authors did include some categories aligning more closely with our readability category, and these were relatively less accurate: while ChatGPT correctly identified 100% of the subject/verb agreement errors in their study, it only identified 39% of the “vocabulary errors” (those occurring in the body of words used in a particular language) [15].

Similar to Lechien et al.’s study [15], Lingard et al. [16] identified that ChatGPT made numerous corrections to their sample of scholarly scientific manuscripts, many neutral or not helpful. The investigators evaluated the corrections, but the authors of the text also reviewed the corrections; many “noted that ChatGPT seemed to ‘fancy up’ their prose, but they were not always sure that this was an improvement.” [16]. The tendency of LLMs to make stylistic edits that may be unnecessary or even detrimental likely stems from the model’s limited contextual nuance and its optimization for general stylistic fluency rather than audience- or domain-specific appropriateness. While some of these edits may improve flow or clarity, others risk introducing artificiality, obscuring meaning, or distancing authors from their authentic voice—particularly in qualitative or narrative-driven research contexts.

This issue was evident in our analysis, where many of U-M GPT’s readability corrections were judged as neutral or worse. Such over-editing may be especially problematic for authors with less confidence in English writing, who may defer to AI suggestions uncritically and adopt language that is overly polished or inadvertently misrepresentative. An additional challenge with U-M GPT is the additional effort required to view corrections as they were not marked in the output and had to be specifically requested.

These findings underscore the need for authors to engage critically with AI-generated edits and highlight the importance of maintaining authorial agency in the editing process. As we emphasize in our conclusion, a higher number of corrections does not equate to better quality, and all AI-generated edits should be carefully reviewed before acceptance.

One question arising from our results is whether authors may learn from LLMs corrections. There is evidence that LLMs can be used to teach writing; [29] however, some have expressed caution about using the technology to support English language writing skills. As Hwang et al. pointed out: “overdependence on LLMs [large language models] may deprive researchers of the opportunity to improve not only their English writing skills but also their understanding of their domain knowledge.” And according to Lingard et al., if ChatGPT is used uncritically, it might actually widen the equity gap in science communication [16]. Thus, the long-term benefits to researchers in using LLMs for English language editing depends on how the technology is used and how critically the output is evaluated by these researchers. An additional concern that could be raised in relation to genAI use is plagiarism; however, the nature of the copyediting performed by ChatGPT is not different than that performed by a human. Human copyediting is not considered plagiarism and therefore, there is no reason to consider it plagiarism when performed by genAI.

Cost is an important consideration and identifying cost-effective editing options was a primary motivation for our research. The cost of a given editor, however, is not simple and can involve hidden costs. Cost is also intertwined with both the time required for editing as well as data privacy affordances. When only considering the amount of money an author would pay for editing, the human was the most expensive option. It took our human editor 3.75 and 4 hours to edit papers 1 and 2, respectively. In the United States, copyeditors charge between $55–70 per hour [30]. We assume that most human editors build data privacy into their service.

Both Grammarly and various LLMs, such as ChatGPT, currently offer free editing services, but they differ significantly in how they handle user data. Grammarly emphasizes user data privacy, claiming that it does not sell or share data with third parties for model training and allows users to opt out of data collection for product improvement. In contrast, OpenAI’s free version of ChatGPT does not guarantee data privacy in the same way, as user interactions are retained for model refinement. However, OpenAI does provide options to enhance privacy, such as disabling chat history, which limits data retention to 30 days and restricts its use to monitoring for misuse rather than for training purposes. Other LLMs, like Anthropic’s Claude (https://claude.ai/), adopt a more privacy-conscious approach, not using user interactions for training and offering automatic deletion within 30 days. Meanwhile, Google’s Gemini retains interactions for up to three years, highlighting the variation in data handling practices across platforms. Privacy is a critical ethical concern because participant data should be kept private and because unpublished manuscript text should not be accessible prior to submission. Thus, while LLMs can democratize access to editing tools, authors must carefully consider the privacy trade-offs of each platform and take advantage of settings that allow greater control over data.

In this study, we used U-M GPT that is secure and private, available through the University of Michigan. While we did not pay to access U-M GPT, this type of closed, secure model is only available to researchers in institutions that can afford them. It is unclear whether the publicly available ChatGPT will remain accessible at no charge. Finally, it is also important to acknowledge that monetary fees are not the only costs associated with genAI use, as there are additional environmental costs due to their enormous energy consumption [31–34]. The environmental toll that technologies such as ChatGPT exact will likely have a greater impact in areas where there is less climate resilience, such as low-income countries [35].

Our author team was surprised to find our sample text blocked by U-M GPT after a request for editing in our pilot study. The response we received (see S1 Text Supporting Information for more details) indicated that the content in the pilot sample paragraphs was filtered. Investigation into U-M GPT’s filtering documentation revealed that the model moderates content that includes words related to women’s health, including anatomical organs, pregnancy, physical sexual acts, and others. Lack of access to copyediting due to content filtering by ChatGPT is a bias that creates inequities that could limit the ability of researchers to promote women’s health and other reproductive and sexual health research. Our study included key strengths. First, we used actual papers written by ESL researchers which adds authenticity to our data and lends credibility and relevance to our findings. Our author team is diverse with the ability to offer perspectives on engaging with an editor as an ESL writer in a low-resource setting.

Our study also has limitations. Our sample included just eight paragraphs and two tables from a total of two manuscripts. The paragraphs were arbitrarily chosen as the second paragraph of each section of the manuscript. These paragraphs and manuscripts do not represent the entire range of typical challenges in global health writing, and the generalizability of this study is limited. A related limitation is that the human editor had access to the entire paper whereas ChatGPT and Grammarly only had access to the paragraphs of interest. Thus, the human editor had the opportunity to understand the broader context of the paper (e.g., the narrative flow, argument structure, and consistent terminology) which inherently informs the editing of any single paragraph. This potential advantage could have created a bias, favoring the human editor; however, the human editor made far fewer edits and all human edits were strictly related to the specific paragraphs under study and none made direct or indirect reference to a different part of the paper in our sample. Another limitation was the subjectivity in quality assessment. It was not possible to create a detailed enough rubric in advance of edit classification as there were too many unique edits. The internal team assessed whether corrections improved, were neutral, or made the text worse, and while the papers were anonymized prior to group review and discussion, the lead author (EA) was aware of the author names. That said, the internal team may have unwittingly introduced bias into the process, for example judging their own human copy editor as improving the text.

To protect the privacy of the authors’ work, we used U-M GPT, which is not publicly available. Our results may not generalize to publicly available genAI models; however, our sensitivity analysis suggested that output from the two versions of ChatGPT were about as similar as those from duplicate requests of U-M GPT. Moreover, given the rapid evolution of genAI technology, the editing landscape is likely to continue changing. Our approach may not have used the most optimized prompts but was designed to mirror realistic usage by researchers. These technologies are still in their infancy, and users are in the early stages of learning how to maximize their potential, for instance, by refining prompt engineering techniques. As genAI tools continue to evolve and improve, and as researchers and other users gain the skills needed to use them effectively, these systems may become increasingly viable and reliable for supporting tasks like copyediting.

## Conclusions

Our investigation evaluated the potential utility of ChatGPT versus Grammarly and a human editor to support authors in creating an acceptable paper for journal peer review. An important aspect of this question is whether technology can support authors with editing in a way that ultimately leads to greater equity. In carrying out this study, we realized just how complicated this question is. While the use of LLMs may provide greater access to a wide scope of editing such as grammar, spelling and suggested revisions to improve readability, we became aware of several ways in which the technology could decrease equity. These include blocking access to editing content related to reproductive health, as well as the complexity of using LLMs for language editing.

Follow-up investigations are needed to confirm our findings. Future research should replicate this study with a larger sample size and variety of manuscript types to strengthen generalizability. Including external reviewers to classify edits and judge them as improving, neutral, or making the text worse would enhance objectivity in further investigating the process through which authors engage with technology to generate, review, and accept corrections.

In the meantime, authors who do use genAI for editing should check a journal’s author guidelines for policies about using and reporting AI and be aware of data privacy concerns. Users should also be aware that genAI copyediting includes risks, including the loss of meaning and readability issues; careful review of each suggested edit is necessary to avoid these problems. A greater number of corrections does not necessarily mean better edits, because many of the edits generated by ChatGPT are neutral or make the text worse. Authors should also be aware of the ethical concerns, including biases in AI filtering.

## Supporting information

S1 Table Number and type of corrections by editor type. (DOCX)

S2 Table Comparison of edits generated from single prompt or prompt chain approaches. (DOCX)

S3 Table Comparison of edits made by the public version of ChatGPT versus U-M GPT. (DOCX)

S1 File Prompts used iteratively in the ChatGPT prompt chain approach. (DOCX)

S2 File Fabricated paragraph for comparison of U-M GPT to the public version of ChatGPT. (DOCX)

S3 File Edits and classifications from the public version of ChatGPT. (DOCX)

S4 File U-M GPT’s first round of edits and classifications. (DOCX)

S5 File U-M GPT’s second round of edits and classifications. (DOCX)

S1 Text Supporting information for methods and materials and detailed results. (DOCX)
