# Supplementary material for: Does ChatGPT enhance equity for global health publications? Copyediting by ChatGPT compared to Grammarly and a human editor
Source: PLoS One. 2026 Feb 5;21(2):e0342170. doi: 10.1371/journal.pone.0342170 (PMC12875453; doi:10.1371/journal.pone.0342170)
Supplement: S1 Table — (DOCX) [file pone.0342170.s001.docx]

**S1 Table. Number and type of corrections by editor type.**

| Type of corrections^a^ | Human  % of total # corrections of a given type (% of total human corrections) | | Grammarly  # corrections of a given type (% of total Grammarly corrections) | | U-M GPT^b^  # corrections of a given type (% of total U-M GPT corrections) | |
| --- | --- | --- | --- | --- | --- | --- |
| Correction Type | Paper 1 | Paper 2 | Paper 1 | Paper 2 | Paper 1 | Paper 2 |
| Total number of corrections per manuscript by editor and correction type | **Total edits: 11**  Introduction: 1  Methods: 5  Results: 0  Discussion: 5 | **Total edits: 22**  Introduction: 8  Methods: 1  Results: 2  Discussion: 11 | **Total edits: 4**  Introduction: 2  Methods: 0  Results: 0  Discussion: 2 | **Total edits: 6**  Introduction: 0  Methods: 2  Results: 1  Discussion: 3 | **Total edits: 51**  Introduction: 16  Methods: 10  Results: 15  Discussion: 10 | **Total edits: 50**  Introduction: 16  Methods: 5  Results: 11  Discussion: 18 |
| Spelling Corrections (including correction of typos) | **Total spelling edits: 0**  Introduction: 0  Methods: 0  Results: 0  Discussion: 0 | **Total spelling edits: 0**  Introduction: 0  Methods: 0  Results: 0  Discussion: 0 | **Total spelling edits: 0**  Introduction: 0  Methods: 0  Results: 0  Discussion: 0 | **Total spelling edits: 1**  Introduction: 0  Methods: 0  Results: 0  Discussion: 1 | **Total spelling edits: 0**  Introduction: 0  Methods: 0  Results: 0  Discussion: 0 | **Total spelling edits: 3**  Introduction: 2  Methods: 0  Results: 0  Discussion: 1 |
| Grammar Corrections (including subject/verb agreement and tense problems) | **Total grammar edits: 3**  Introduction: 0  Methods: 2 (check)  Results: 0  Discussion: 1 | **Total grammar edits: 2**  Introduction: 0  Methods: 0  Results: 0  Discussion: 2 | **Total grammar edits: 2**  Introduction: 1  Methods: 0  Results: 0  Discussion: 1 | **Total grammar edits: 2**  Introduction: 0  Methods: 1  Results: 1  Discussion: 0 | **Total grammar edits: 1**  Introduction: 1  Methods: 0  Results: 0  Discussion: 0 | **Total grammar edits: 0**  Introduction: 0  Methods: 0  Results: 0  Discussion: 0 |
| Punctuation, Spacing, and Capitalization Corrections | **Total punctuation, spacing, and capitalization edits: 2**  Introduction: 0  Methods: 2  Results: 0  Discussion: 0 | **Total punctuation, spacing, and capitalization edits: 2**  Introduction: 0  Methods: 1  Results: 1  Discussion: 0 | **Total punctuation, spacing, and capitalization edits: 0**  Introduction: 0  Methods: 0  Results: 0  Discussion:0 | **Total punctuation, spacing, and capitalization edits: 0**  Introduction: 0  Methods: 0  Results: 0  Discussion: 0 | **Total punctuation, spacing, and capitalization edits: 6**  Introduction: 2  Methods: 1  Results: 3  Discussion: 0 | **Total punctuation, spacing, and capitalization edits: 8**  Introduction: 3  Methods: 2  Results: 3  Discussion: 0 |
| Unclear text flagged | **Unclear text flagged: 4** 1 instance from methods, 2 instances from the discussion and one instance from Table 2 | **Unclear text flagged: 3** 1 instance from the introduction and 2 instances from the discussion | **Unclear text flagged: 0** | **Unclear text flagged: 0** | **Unclear text flagged: 0** | **Unclear text flagged:0** |
| Corrections to improve readability, flow, or style | **Total readability, flow, style edits: 6**  Introduction: 1  Neutral: 0  Improved: 1  Made worse: 0  Of style changes, how many changed meaning? 0  Methods: 1  Neutral: 1  Improved: 0  Made worse: 0  Of style changes, how many changed meaning? 0  Results: 0  Neutral: 0  Improved: 0  Made worse: 0  Of style changes, how many changed meaning? 0  Discussion: 4  Neutral: 0  Improved: 3  Made worse: 1  Of style changes, how many changed meaning? 0 | **Total readability, flow, style edits: 15**  Introduction: 7  Neutral: 0  Improved: 7  Made worse: 0  Of style changes, how many changed meaning? 0  Methods: 0  Neutral: 0  Improved: 0  Made worse: 0  Of style changes, how many changed meaning? 0  Results: 1  Neutral: 0  Improved: 1  Made worse: 0  Of style changes, how many changed meaning? 0  Discussion: 7  Neutral: 0  Improved: 7  Made worse: 0  Of style changes, how many changed meaning? 0 | **Total readability, flow, style edits: 2**  Introduction: 1  Neutral: 0  Improved: 1  Made worse: 0  Of style changes, how many changed meaning? 0  Methods: 0  Neutral: 0  Improved: 0  Made worse: 0  Of style changes, how many changed meaning? 0  Results: 0  Neutral: 0  Improved: 0  Made worse: 0  Of style changes, how many changed meaning? 0  Discussion: 1  Neutral: 1  Improved: 0  Made worse: 0  Of style changes, how many changed meaning? 0 | **Total readability, flow, style edits: 3**  Introduction: 0  Neutral: 0  Improved: 0  Made worse: 0  Of style changes, how many changed meaning? 0  Methods: 1  Neutral: 1  Improved: 0  Made worse: 0  Of style changes, how many changed meaning? 0  Results: 0  Neutral: 0  Improved: 0  Made worse: 0  Of style changes, how many changed meaning? 0  Discussion: 2  Neutral: 1  Improved: 1  Made worse: 0  Of style changes, how many changed meaning? 0 | **Total readability, flow, style edits: 44**  Introduction: 13  Neutral: 3  Improved: 7  Made worse: 3  Of style changes, how many changed meaning? 3  Methods: 9  Neutral: 2  Improved: 5  Made worse: 2  Of style changes, how many changed meaning? 1  Results: 12  Neutral: 2  Improved: 9  Made worse: 1  Of style changes, how many changed meaning? 1  Discussion: 10  Neutral: 2  Improved: 8  Made worse: 0  Of style changes, how many changed meaning? 0 | **Total readability, flow, style edits: 39**  Introduction: 11**  Neutral: 2  Improved: 7  Made worse: 2  Of style changes, how many changed meaning? 2  Methods: 3  Neutral: 0  Improved: 3  Made worse: 0  Of style changes, how many changed meaning? 0  Results: 8  Neutral: 3  Improved: 3  Made worse: 2  Of style changes, how many changed meaning? 0  Discussion: 17  Neutral: 6  Improved: 9  Made worse: 2  Of style changes, how many changed meaning? 3 |
| Key information Removed^c^ |  |  |  |  |  | Total: 10  Introduction: 6 references removed  Results:  reference to Table 2 removed  Discussion: 2 references removed |

^a^Adapted from classifications by Park et al^4^

^b^The approach used was a single prompt approach

^c^The need for this category was not anticipated prior to analysis and was added at the time of analysis; the 10 instances of key information that was removed is not counted in the tally because the category was created in an ad hoc fashion, during analysis.
