# Supplementary material for: Does ChatGPT enhance equity for global health publications? Copyediting by ChatGPT compared to Grammarly and a human editor
Source: PLoS One. 2026 Feb 5;21(2):e0342170. doi: 10.1371/journal.pone.0342170 (PMC12875453; doi:10.1371/journal.pone.0342170)
Supplement: S2 Table — (DOCX) [file pone.0342170.s002.docx]

**S2 Table.** **Comparison of edits generated from single prompt or prompt chain approaches**.

|  | Single Prompt^a^ | Prompt Chain^b^ | Percent agreement |
| --- | --- | --- | --- |
| Spelling Corrections (including correction of typos) | 0 | 0 | 100% |
| Grammar Corrections (including subject/verb agreement, tenses) | 0 | 0 | 100% |
| Capitalization | 0 | 0 | 100% |
| Punctuation | 0 | 0 | 100% |
| Corrections to improve readability, flow, or style | Total: 10  Neutral: 2  Improved: 8  Made worse: 0  Of style changes, how many changed meaning? 0 | Total: 11  Neutral: 2  Improved: 8  Made worse: 1  Of style changes, how many changed meaning? 1 | 9/13=69%§ |
| Flag unclear text | 0 | 0 | 100% |
| Total edits | 10 | 11 |  |

^a^Shown in Box in main paper

^b^Shown in Box S1
