# Supplementary material for: Does ChatGPT enhance equity for global health publications? Copyediting by ChatGPT compared to Grammarly and a human editor
Source: PLoS One. 2026 Feb 5;21(2):e0342170. doi: 10.1371/journal.pone.0342170 (PMC12875453; doi:10.1371/journal.pone.0342170)
Supplement: S3 Table — (DOCX) [file pone.0342170.s003.docx]

**S3 Table. Comparison of edits made by the public version of ChatGPT versus UM-GPT.**

|  | Public version | U-M GPT (round 1) | U-M GPT (round 2) | All three edits identical | Public and first round of U-M GPT edits identical | Public and second round of U-M GPT edits identical | First and second round of U-M GPT edits identical | Average # edits across three requests |
| --- | --- | --- | --- | --- | --- | --- | --- | --- |
| Added word or phrase | 6 | 7 | 4 | 2 | 2 | 2 | 4 | 5.7 |
| Deleted word or phrase | 1 | 1 | 1 | 1 | 1 | 1 | 1 | 1 |
| Revise word or phrase | 16 | 16 | 15 | 1 | 1 | 1 | 5 | 15.7 |
| Punctuation and spacing changes | 5 | 3 | 5 | 3 | 3 | 3 | 3 | 4.3 |
| Capitalization | 0 | 1 | 1 | 0 | 0 | 0 | 1 | 0.7 |

All edits were generated on the same day (May 19, 2025).
