# Supplementary material for: Does ChatGPT enhance equity for global health publications? Copyediting by ChatGPT compared to Grammarly and a human editor
Source: PLoS One. 2026 Feb 5;21(2):e0342170. doi: 10.1371/journal.pone.0342170 (PMC12875453; doi:10.1371/journal.pone.0342170)
Supplement: S1 File — (DOCX) [file pone.0342170.s004.docx]

**S1 Box. Prompts used iteratively in the ChatGPT prompt chain approach.**

| 1. Copy edit this text from a scientific journal article so that it can be submitted to a peer-reviewed journal. Correct spelling errors and other types of errors. 2. Copy edit this text from a scientific journal article so that it can be submitted to a peer-reviewed journal. Correct grammatical errors and other types of errors. 3. Copy edit this text from a scientific journal article so that it can be submitted to a peer-reviewed journal. Correct capitalization and other types of errors. 4. Copy edit this text from a scientific journal article so that it can be submitted to a peer-reviewed journal. Correct tenses and other types of errors. 5. Copy edit this text from a scientific journal article so that it can be submitted to a peer-reviewed journal. Correct typos and other types of errors. 6. Copy edit this text from a scientific journal article so that it can be submitted to a peer-reviewed journal. Correct punctuation and other types of errors. 7. Copy edit this text from a scientific journal article so that it can be submitted to a peer-reviewed journal. Correct subject/verb agreement and other types of errors. 8. Copy edit this text from a scientific journal article so that it can be submitted to a peer-reviewed journal. Revise to improve clarity, readability, flow, and style, however, if the meaning of the text is unclear then do not attempt to revise the text, instead state that the text is unclear. Correct other types of errors. |
| --- |
