# Supplementary material for: Does ChatGPT enhance equity for global health publications? Copyediting by ChatGPT compared to Grammarly and a human editor
Source: PLoS One. 2026 Feb 5;21(2):e0342170. doi: 10.1371/journal.pone.0342170 (PMC12875453; doi:10.1371/journal.pone.0342170)
Supplement: S2 File — (DOCX) [file pone.0342170.s005.docx]

**S2 Box. Fabricated paragraph for comparison of U-M GPT to the public version of ChatGPT.**

| Adolescents 10-19 make nearly 25% of the population in Zambia. one in four adolescents younger than 20 have a child or pregnant. One fourth of women 25-49 report sexual debut before 16th birthday, and one half before 18. The average of the lag between first sex and first contraceptive use is more than five years. Thus, many Zambian women start sex at adolescence, but do not use a contraceptive when they first start having sex. This mismatch can be from a lack of access for contraceptives and a stigma for buying contraceptives. |
| --- |
