# Supplementary material for: Does ChatGPT enhance equity for global health publications? Copyediting by ChatGPT compared to Grammarly and a human editor
Source: PLoS One. 2026 Feb 5;21(2):e0342170. doi: 10.1371/journal.pone.0342170 (PMC12875453; doi:10.1371/journal.pone.0342170)
Supplement: S3 File — (DOCX) [file pone.0342170.s006.docx]

**S3 Box.** **Edits and classifications from the public version of ChatGPT.**

| Adolescents aged [add word/phrase]10–19 [punctuation/spacing] comprise [revise word/phrase] nearly 25% of the [punctuation/spacing] population [punctuation/spacing] in Zambia. One in four adolescent girls under the age of 20 is either pregnant or already has a child [revise word/phrase]. Among [add word/phrase] women aged [add word/phrase] 25–49[punctuation/spacing] , one in four reports [revise word/phrase] having initiated [add word/phrase] sexual activity [revise word/phrase] before the age of 16 [revise word/phrase], and [delete word/phrase] half report doing so [add word/phrase] before the age of [add word/phrase] 18. The average time [revise word/phrase] between sexual debut [revise word/phrase] and first use of contraception exceeds [revise word/phrase] five years.  These findings suggest that [revise word/phrase] many Zambian women initiate sexual activity during [revise word/phrase] adolescence [punctuation/spacing] but do not use contraception at the time of sexual debut. [revise word/phrase] This gap may [revise word/phrase] be attributed to limited [revise word/phrase] access to [revise word/phrase] contraceptives and social [revise word/phrase] stigma associated with obtaining them. [revise word/phrase] |
| --- |
