# Supplementary material for: Does ChatGPT enhance equity for global health publications? Copyediting by ChatGPT compared to Grammarly and a human editor
Source: PLoS One. 2026 Feb 5;21(2):e0342170. doi: 10.1371/journal.pone.0342170 (PMC12875453; doi:10.1371/journal.pone.0342170)
Supplement: S4 File — (DOCX) [file pone.0342170.s007.docx]

**S4 Box. U-M GPT’s first round of edits and classifications.**

| Adolescents aged [add word/phrase] 10-19 constitute [revise word/phrase] nearly 25% of the [punctuation/spacing] population [punctuation/spacing] in Zambia. One [capitalization] in four adolescents younger than 20 either has [revise word/phrase] a child or is [add word/phrase] pregnant. A quarter [revise word/phrase] of women aged [add word/phrase] 25-49 report having their [add word/phrase] sexual debut before their [add word/phrase] 16th birthday, and [delete word/phrase] half before reaching [add word/phrase] 18. On [revise word/phrase] average, there is a [revise word/phrase] lag of more than five years [revise word/phrase] between a woman's [add word/phrase] first sexual experience [revise word/phrase] and first [revise word/phrase] use of contraceptives. Consequently, [revise word/phrase] many Zambian women begin sexual activity in [revise word/phrase] adolescence [punctuation/spacing] but do not use contraceptives initially. [revise word/phrase] This mismatch may [revise word/phrase] be due to [revise word/phrase] a lack of access to [revise word/phrase] contraceptives and the [revise word/phrase] stigma  associated with purchasing them. [revise word/phrase] |
| --- |
