# Supplementary material for: Does ChatGPT enhance equity for global health publications? Copyediting by ChatGPT compared to Grammarly and a human editor
Source: PLoS One. 2026 Feb 5;21(2):e0342170. doi: 10.1371/journal.pone.0342170 (PMC12875453; doi:10.1371/journal.pone.0342170)
Supplement: S5 File — (DOCX) [file pone.0342170.s008.docx]

**S5 Box. U-M GPT’s second round of edits and classifications.**

| Adolescents aged [add word/phrase] 10 to [punctuation/spacing] 19 constitute [revise word/phrase] nearly 25% of the [punctuation/spacing] population [punctuation/spacing] in Zambia. One [capitalization] in four adolescents younger than 20 is either pregnant or has [revise word/phrase] a child. Approximately one-[revise word/phrase] fourth of women aged [add word/phrase] 25 to [punctuation/spacing] 49 report having their [add word/phrase] sexual debut before their [add word/phrase] 16th birthday, and [delete word/phrase] half before 18. The average gap [revise word/phrase] between first sexual intercourse [revise word/phrase] and initial [revise word/phrase] contraceptive use is more than five years.Consequently, [revise word/phrase] many Zambian women begin sexual activity during [revise word/phrase] adolescence [punctuation/spacing] but do not use contraceptives [revise word/phrase] when they first become sexually active. [revise word/phrase] This discrepancy may [revise word/phrase] be due to limited [revise word/phrase] access to [revise word/phrase] contraceptives and the [revise word/phrase] stigma  associated with purchasing them. [revise word/phrase] |
| --- |
