# Supplementary material for: Does ChatGPT enhance equity for global health publications? Copyediting by ChatGPT compared to Grammarly and a human editor
Source: PLoS One. 2026 Feb 5;21(2):e0342170. doi: 10.1371/journal.pone.0342170 (PMC12875453; doi:10.1371/journal.pone.0342170)
Supplement: S1 Text — (DOCX) [file pone.0342170.s009.docx]

**Pilot study methods**

We started our investigation with a pilot study in April of 2024 with two draft papers: one from Ethiopia and one from Uganda drawn from the group of east African researchers who were part of the CIRHT/PREPSS training program identified in the main text.(1–3) The goal of the pilot study was to test our draft protocol, and to identify and resolve any problems or oversights before starting the main study.

To maintain data privacy, we used U-M GPT, a secure, University of Michigan-hosted generative AI tool (<https://umgpt.umich.edu/>). While U-M GPT does not charge users, the institution bears cost in implementing and maintaining this closed system. Our original U-M GPT prompt was: “Copy edit this scientific journal article so that it can be submitted to a peer-reviewed journal called [stated the target journal here, for example Reproductive Health Journal]. Correct spelling errors, grammatical errors, capitalization, tenses, typos, punctuation, subject/verb agreement and other types of errors. Also please revise for clarity.”

We learned several things about using U-M GPT from the pilot study. First, we were surprised that the software initially refused to edit the methods, results, and discussion sections of the pilot papers. U-M GPT generated the following output explaining why it would not respond to our prompt: “Your request generated an invalid response. Invalid responses may be caused by content filtering. Please try again.” Though U-M GPT did not point to specific words as activating its content filter, we speculated that words such as “sterilization” and “abortion” likely triggered it, initially blocking our ability to edit the paper.^[[1]](#footnote-1)^ Upon further investigation, we identified that “sexual content” is subject to moderation policies that restrict discussions on topics deemed as sensitive by U-M GPT. The documentation, provided by Azure OpenAI service, states: “Sexual describes language related to anatomical organs and genitals, romantic relationships, acts portrayed in erotic or affectionate terms, pregnancy, physical sexual acts, including those portrayed as an assault or a forced sexual violent act against one’s will, prostitution, pornography, and abuse.”

We also found that including the name of the author’s target journal caused the output to be too focused on formatting advice which was not the goal of this analysis, so we removed this part of the prompt in the main study. We also revised the prompt to be more specific to the editing categories we identified for analysis (shown in Table S1; see final prompt in the main study methods section) and to match the guidance provided to the human copy editor.

**Detailed results from main study**

The eight paragraphs contained 898 words, with 436 words from Paper 1 and 462 words from Paper 2. The introduction sections contained 137 and 140 words, from Papers 1 and 2 respectively, the methods contained 99 and 56 words, respectively, the results included 82 and 111 words, respectively and the discussion contained 118 and 155 words, respectively. A breakdown of the corrections from each editor by paper and section are provided in Table S1.

**Sensitivity analyses**

**Single prompt versus prompt chaining**

Large Language Models (LLMs) experts advise avoiding complex prompts because the model may struggle to address multiple prompts at the same time. We included a single prompt (described above) to align with our goal of testing the feasibility of using LLMs for copyediting under real world conditions. However, to identify potential limitations in our single-prompt approach, we conducted a sensitivity analysis that compared the results to those generated from a prompt chain approach, wherein a bigger prompt is broken into smaller, focused sub-prompts to our single prompt approach.

To carry out the prompt chaining approach, the text from one discussion paragraph from Paper 1 was evaluated iteratively eight times using the prompts in Box S1. Copyedits were generated by evaluating the output from the first prompt and implementing appropriate corrections, including readability, flow, and style corrections that were judged by us as neutral. Once the suggested corrections were either rejected or accepted, the revised paragraph was evaluated by the second prompt, and so on until the paragraph was evaluated by all eight prompts.

The final output with corrections from the eighth prompt were compared to the corrections from the single-prompt approach. A comparison of the number and type of edits were calculated, along with the percent agreement between the two approaches. Readability corrections from the single prompt versus prompt chain approach were counted as the same if the changes were similar in wording and were both considered to improve, worsen, or have a neutral impact on the text.

There was 100% agreement between the single prompt and prompt chain approaches for spelling corrections (including correction of typos), grammar corrections (including subject/verb agreement, tenses), capitalization, punctuation, and flagging unclear text categories (Table S2, Supporting Information). There were differences in the readability, flow, or style between the two approaches. Collectively, there were a total of 13 unique readability corrections across the prompt chain and single prompt approaches (not shown), and nine of these (69%) agreed (Table S2). Both the prompt chain and single prompt approach suggested 8 corrections deemed to improve the text, and both also suggested two neutral corrections. However, the prompt chain approach also provided one correction that worsened the quality of the text, whereas the single-prompt approach did not suggest any such corrections.

Four readability, flow, and style corrections differed between the single and prompt chain approaches. Two of the four corrections were judged as neutral (one from the single prompt approach did not appear in the prompt chain approach and the other was generated in the prompt chain but did not appear in the single prompt approach; not shown). The other two unique corrections were both generated from the prompt chain approach with no analogous corrections in the single prompt approach. In the first, the correction was judged to worsen the original text; and in the second, the correction was judged to improve the text.

**Comparison of U-M GPT with the public version of ChatGPT**

To compare edits from the public version of ChatGPT (GPT-4, the GPT-4o "omni" model) with U-M GPT, we requested copyedits from both on the same day using the single prompt from our main analysis. We fabricated a paragraph to sound roughly like text from a global health research manuscript written by a second language speaker, intentionally adding language errors (Box S2). To address whether and how much output varied within the same version, we also asked U-M GPT to edit the prompt a second time, immediately after the first request.

The edits generated from the public version of ChatGPT are shown in Box S3, and those generated from the first and second round of requests from U-M GPT are shown in Boxes S4 and S5, respectively with track changes. We classified the edits into five categories, including adding a word or phrase, deleting a word or phrase, revising a word or phrase, punctuation and spacing changes, and capitalization and the classifications are shown in brackets after each edit in Boxes S3-S5.

A summary table compares the number of edits in each of the five categories (Table S3). For the added word or phrase category, each editor made a different number of edits. All three editors made two identical edits in this category, but the first and second round of U-M GPT edits shared four identical added word or phrase suggestions. All editors suggested deleting the same word. The public version of ChatGPT did not capitalize any text, but the first and second round of U-M GPT edits both capitalized the same word. For suggested word or phrase revisions, the public version and first round of U-M GPT edits both suggested 16 edits and the second round of U-M GPT suggested 15 edits. While only one of these edits was identical across all three editors, many of them were very similar (e.g., a suggestion to change “make” to “comprise” versus “constitute”). Five identical word or phrase revisions were shared across the first and second round of U-M GPT. Finally, both the public version and second round of U-M GPT suggested 5 punctuation and spacing edits but the first round of U-M GPT produced 3 edits. Three of these edits were identical across editors.

**References**

1. Buser JM, Tengera O, Jiang C, Kumakech E, Gray R, Mukeshimana M, et al. Impact of a comprehensive two-year intervention on research competence in Sub-Saharan Africa: A pre-post training evaluation using the CRAI-12 scale. Reprod Health.

2. Busse C, August E. Addressing power imbalances in global health: Pre-Publication Support Services (PREPSS) for authors in low-income and middle-income countries. BMJ Glob Health. 2020 Feb;5(2):e002323.

3. Buser JM, Morris KL, Millicent Dzomeku V, Endale T, Smith YR, August E. Lessons learnt from a scientific peer-review training programme designed to support research capacity and professional development in a global community. BMJ Glob Health. 2023 Apr;8(4):e012224.

1. We identified additional information about content filtering at this link (U-M GPT is consuming Azure OpenAI services): <https://learn.microsoft.com/en-us/azure/ai-services/openai/concepts/abuse-monitoring> and <https://learn.microsoft.com/en-us/azure/ai-services/openai/concepts/content-filter?tabs=warning%2Cpython-new>. [↑](#footnote-ref-1)
